# Are we really surmounting the binary? Visualizing race and ethnicity group relations via embedded relational diagrams

**DOI:** 10.3389/fsoc.2025.1553566

**Published:** 2025-04-02

**Authors:** Rima Wilkes, Aryan Karimi

**Affiliations:** Department of Sociology, University of British Columbia, Vancouver, BC, Canada

**Keywords:** visualization, social theory, theoretical diagrams, racial theories, assimilation theories

## Abstract

Social theories explain the current state of affairs between social groups. In the sociological race literature, theories traditionally explained White-Black relations. In the ethnicity literature, theories explained native-born and immigrant relations. What happens to social theorizing of current group relations when new third groups emerge in society? Because social reconfigurations unfold in the longue durée and are less amenable to controlled tests, social sciences are still in the process of theorizing the effects of third groups on the old racial and ethnic relations. To outline the theorizing process, its elements, and its challenges, we propose a novel embedded relational visual diagram of the triadic relationship between Asian American, Black American, and White American groups. We use a range of six theories from the race and ethnicity literature as case studies to illustrate the applicability of the visualizing method. We show why these triadic social relations inevitably collapse into a new social duality.

## Introduction

In everyday life, human cognition perceives the social world though an us-them lens wherein the “us” group has some form of consistent relation with the “them” individuals or groups (Barth, 1969; Cerulo et al., 2021; Lamont et al., 2014). It is true that each of the us-them groups has infinite internal diversities, but when pushed to the extremes, these diversities coalesce behind the cognitive us-them boundaries. In the social sciences, there is a tendency is to theorize futures where the old duality is undone and new diversities materialize. This is often done by creating a continuum and scattering the new groups along the two ends which represent the old two groups; adding new concepts or categorization for the third group such as “ethnoracial”, “fluidity”, “intersectional”, “hybrid”, “liminal”, “localized”, “mixed-race”, “multiracial”, “overlapping”, “shifting”, “transcendent”, “transnational”; or by using some combination of these strategies (Brubaker and Fernández, 2019; Brunsma et al., 2013; Cornell and Hartmann, 2006; Bynner, 2005; Modi, 2023; Valdez and Golash-Boza, 2017; Wacquant, 2024).

The logic is effectively that the third group transcends the duality and hence occupies a positionality that requires these other concepts. Fundamentally, in relation to the “either/or” logic of the duality, these third groups entail an “and/both” or a “neither/nor” categorization. Theorizings of diverse and egalitarian futures based on third groups are buttressed by sophisticated (methodological) interventions such as such as fuzzy-set (Bail, 2008; Monk, 2022) and ordinalization analyses (Fourcade, 2016) both of which, it is said, surmount the pre-existing categorizations. Then, it appears that the theory and its society no longer reference a duality but a multiplicity of super diversities (Vertovec, 2007, 2019) and hyper-mobilities (Guo, 2022).

Still, consider the social world from the first-person perspective of the individual or group rather than from the theoretician's third-person perspective. Take the race example. If the old duality or binary in the U.S., from the individual perspective, had been White-Black, recent demographic changes and, hence, the race literature now adds a new “mixed-race” category to opine for a multiracial future. Unless every single individual inter-marries and, say in the next millennium, we are all mixed-race, the empirical reality remains that, from the perspective of the existing mixed-race individuals, the social world often has an us-them duality in the form of mixed-race vs. non-mixed-race groups. If the mixed-race individuals transcend the categories of the old “Black” or “White” race duality, a new “single” or “mixed” duality then emerges. While a “single” or “mixed” race duality is “post” and transcendent of the old Black or White racial duality this new duality is not “post” duality writ large. The same logic applies to other new categories such as superdiverse or intersectional (vs. non-intersectional), fluid/shifting (vs. non-fluid/fixed), transnational (vs. non-transnational), and hybrid (vs. non-hybrid) since these former categories are not universal and are applied to a subset of social groups. In all these comparisons the contrast with the new third group as “post” then also serves to push the groups from the old duality together.

In this paper, we illustrate that, at a fundamental level, social sciences' three-body problem is not much different from the natural sciences' 300-year-old challenge of navigating phenomenological dualities.^1^ Given that the three-body problem of three collapsing into two remains unsolved in physics, it is improbable that the solution is a simple matter of movement of third groups into different positionalities. Indeed, because social changes unfold in the longue durée, outside of lab-like controlled environments, or perhaps due to Hawthorne effect (or observer bias), the observation that three or more social groups ultimately collapse into two is masked but not solved.

To uncover the challenges of theorizing the three-group situation, we focus on particular theories from the race and ethnicity (assimilation) paradigms as case studies. We follow the U.S. lens on race and ethnicity (for the European approach see Wimmer, 2013; Wacquant, 2024). We use a range of six theories which are eminent and represent the dynamism of the race and ethnicity paradigms. These include theories of White supremacy (Christian, 2019; Feagin and Elias, 2013; Ren and Feagin, 2021), racial triangulation (Aronson and Stohry, 2023; Kim, 1999; Wong and Ramakrishnan, 2023), anti-Blackness (Davies, 2022; Kim, 2023; Sexton, 2010), as well as segmented assimilation (Portes and Zhou, 1993); ethnic economy (Light et al., 1994), and neo-assimilation (Alba and Nee, 1997, 2003).^2^

We develop a new embedded relational method of visual diagramming the triadic relationship between the Asian American, Black American, and White American groups. Visual articulations of theory continue to be a relative rarity in the race and assimilation literatures and a growing body of literature suggests that visual representation is important not only for representation and illustration but also for theoretical development (Brett et al., 2020; Brett and Silver, 2024; Silver, 2020; Swedberg, 2016). The visualizations portray the old duality and the three-group relations side by side. This visual comparison allows for intuitively illustrating the emerging duality among the three groups in ways that may be less apparent with text alone.

The aim is to describe how each of these theories is delineating the three-group situation. We do not test and/or advocate for a particular theory or paradigm and acknowledge that some or all of these theories or their categorization of particular groups may be viewed as contentious. Within each paradigm and theory, for consistency and comparative analyses, we use the same groups of White Americans and Black Americans and the third group of Asian Americans—the group choice is *ad hoc* and can be replaced by Latino or Middle-Eastern Americans, among others.

In the following sections, we begin by outlining our proposed visualization method, and proceed to discussing and visualizing the race and ethnicity paradigms. We show that the three-group versions of each of the theories still reproduce as a new two-group duality. Race theories' duality is about groups' positions vs. one another while the ethnicity theories' duality is about which group experiences SES mobility and groups' do not experience SES mobility over time. Accordingly, the white supremacy and racial triangulation models reproduce a White—non-White perspective and anti-Blackness a Black—non-Black view while the ethnicity (assimilation) theories reproduce an Asian vs. non-Asian duality whereby assimilation and upward mobility is expected from Asian American but not the White and Black American groups. We then consider the theoretical implications of a change to the triadic group rankings in the Discussion section. Whether by including or excluding the new third group, human cognition and its theories appear to construct new us-them boundaries (Barth, 1969).

## Embedded-relational visualization method

The core objective is to intuitively visualize multiple race and assimilation theories via graphs that use simple XY axes and a few arrows. The underlying value of this method is its capacity to condense manifold textual descriptions of theories into coherent graphs and to facilitate comparative analyses across numerous social theories. We call the method “embedded relational” insofar as it embeds relationships within the XY axes. The embedded relational graphing approach meets the standard for “theorizing diagrams” insofar as the “diagram should also be able to guide you in certain directions without locking you into a single solution” (Swedberg, 2016, p. 259).

At its most basic form, a social theory outlines an observable relationship between a minimum of two entities such as proletariat-bourgeois or White-Black. The relationship is a meaning or value system is a matter of consensus and impositions and is between a minimum of two groups such as oppressed-oppressor or superior-inferior. Because there is one concept, such as class or race, and two groups within the concept, the relationship between the two groups remains descriptive and limited to their status quo. The goal of theorizing, across sciences, is to make predictions about future outcomes that go beyond the status quo (Popper, 2002 [1935]; Hempel, 1965). Predictions occur when a new group (within the same concept) or a totally new concept appears on the empirical scene. The appearance is either artificial or natural; that is, either the researcher adds a third entity to the calculations or that a third entity organically shows up and compels the researcher to contend with its effect. Either way, this is how theories become dynamic and future-oriented so as to build better hypotheses and better societies (see Fuhse, 2022).

For instance, in class relations between proletariat-bourgeois, the status quo description was that the latter oppresses the former. Similarly, in race relations between the White-Black groups, the status quo description was that the former oppresses the latter. But what happens when the uber wealthy owners of Amazon and SpaceX appear, or when Asian (and other) immigrant groups arrived in the U.S. in large numbers? The addition of these third groups throws the exiting dual relations off balance by forcing a question: how does this third group change the existing relations in the future?

Accordingly, each dynamic future-oriented theory requires several components to calculate and predict the forms of new group positions and their social relations. The primary analytical challenge is to distinguish the concepts, their categories, and the groups which are then attached to each of the categories (see Abend, 2019 on the contents of “thick concepts”). As Loveman (1999) notes, these analytical steps must be disentangled such that they are not treated as one and the same in, for instance, the case of race: concept (race) = category (Black Am.) = group (Black Am.). This lack of distinction between the elements shows that the concept, categories, and groups are essentialized and, hence, devoid of theoretical and social dynamism. With this in mind, we underline the elements of a social theory as follows:

- Concepts: these are general abstract ideas or words used to describe the world. Examples include race, ethnicity, class, sexuality, time, and age. In embedded relational visualizations, the concepts are the XY axes or dimensions of the graph.
- Categories: these are socially arbitrary but conventional positions within a given concept—meaning that these positions are fluctuating social constructs. Each concept has a minimum of two categories within it—with only category, the category and concepts become essentialized. Historically, examples of such categories or values include superior-inferior for race and wealthy-poor for class. In social theorizing, and in us-them identifications, categories are then the site of social struggle and, as such, are imbued with values.
- In embedded relational graphs, categories denote the endpoints of each axis. It is possible to theorize more nuanced gradations and categories in the middle of the continuum but from the perspective of the individual on the end of the continuum, the next person on the continuum is the “them” individual. From the perspective of the individual in the middle, either/both ends of the continuum are the “them” individual. No matter how fine-grained of a continuum, the first-person perspective reproduces the us-them duality (Karimi and Wilkes, 2025; Tilly, 1998; Wilkes and Karimi, 2023).^3^
- Groups: these are social groups such as White-Black, bourgeoisie-proletariat, and male-female. In the graphs, each group is assigned to one category in each concept and, as a result of this cross-classification, the group occupies one spot along the XY axes. Again, these categorical assignations are fluid and contain both self- or other-ascriptions (Barth, 1969; see Brubaker, 2004 category of practice vs. category of analysis). Examples include the affixation of the White-Black groups to the superior-inferior categories for the race concept and the affixation of the bourgeois-proletariat groups to the wealthy-poor categories for the class concept.^4^
- Relationships: these are the ways that groups see one another from their own first-person perspectives. Examples include the class oppression relation where the proletariat sees itself as oppressed and the bourgeois as the oppressor. In embedded relational visualizations, a relation is represented by an arrow. If groups are placed in the same spot on the XY axes, then they are in the same category and lack an inter-categorical relation. The presence of these (relationship) arrows between groups hints at why categorization is contentious (see Barth, 1969 on this point).

Overall, the visualizations comprehensively encompass these four theoretical elements. Then, to create a graph, the process of outlining all of the elements of a theory, concept by concept, should be explicitly identified. We call these graphs embedded relational graphs for the following reason: To connect the visualization method with the routine methods of creating 2 × 2 tables in preparation for noting the appropriate equations for data analyses, we underline that the visualization approach combines the 2 × 2 table and group-arrow (network-relational) approaches to hypothesis generation. In effect the embedded relational diagrams combine theoretical structural geometries or what have been called “Parsons four-field schemes” and “Levi-Strauss triangles” (Guggenheim, 2024, p. 179).

The 2 × 2 table approach takes the concepts and their categories and places the groups in quadrants of the table. Because this is the last step of creating a 2 × 2 table, the relationships between the groups, implied by their relative placement in the quadrants, are omitted. By contrast, the group-arrow approach entails placing the groups spatially and delineating their relationships (from whom to whom) via arrows. This is also the logic underlying network diagrams. The group-arrow approach omits the concepts and their categories. Instead, the embedded relational approach overcomes both limitations by placing the group-arrows within the XY axes which leads to an accounting of the group positions and a more accurate accounting of all possible relationships between the groups.

**Figure 1:**
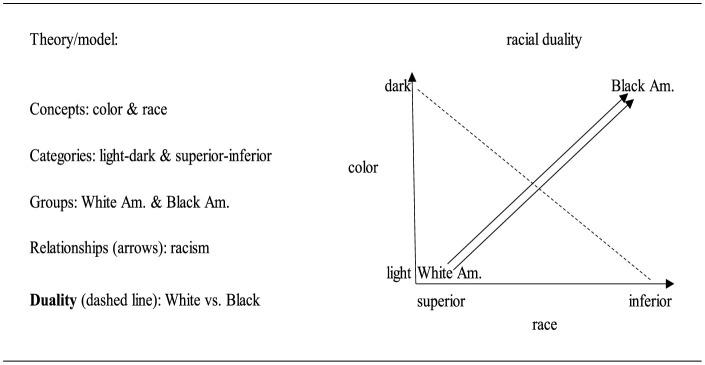
The main components of the original race duality in embedded relational visualization.

Returning to the main goal of this paper, which is to breakdown and visualize the race-assimilation paradigms and their theories, Figure 1 applies the embedded relational visualization method to the original arguments that propagated the current (critical) race paradigm.^5^

Figure 1 reflects the original idea of racial thinking in the U.S. and the theoretical elements which shaped it. The graph shows two arrows between White and Black Americans delineating racism as multidimensional. Undergirding the critical race paradigm is an understanding of the arrows as delineating forms of racism. While other paradigms might understand the arrows to have a meaning other than racism each arrow can only ever reflect a single concept. We also note the direction of the X (race) and Y (color) axes delineated by arrows. As per standard graphical practices, the zero point is where the arrows meet with the bottom to up and left to right delineating a movement from 0 to 1 (racism). Without these arrows, racial groups and their positions are reflected in the quadrants but not the relationship between them. While it has been common to represent White supremacy such as spatially top-down or in a pyramidal manner (e.g., see Christian, 2019) with the arrows going in a different direction than shown here, the axes of the graph that determine the direction of the relationship. Thus, since the direction goes from the meeting of the axes outwards, the position of the different groups must be organized accordingly. The arrows comprising the axes are theorized as delineating racism and then the arrows within the graph (also delineating racism) should follow this direction. That said, even if the positions of the White and Black groups were reversed such that White was on the visual top, the number of arrows between the groups, and hence the implications, would not change.

Historically, religion had shaped group boundaries in Europe. The main duality was Christians vs. non-Christians with the latter entailing another duality within in it, Moors and Jews (Heng, 2011; see Wacquant, 2024, p. 48 on three roots of race in Europe). With the advent of imperialism and colonialism across the world at the time of Enlightenment and scientific thinking, Enlightenment thinkers assimilated the previous cultural-religious categories to develop new categories (Arendt, 1944; Greer et al., 2008; Painter, 2010). As early as the eighteenth century, European social scientist and philosophers perceived a new *color* line division which divided the world's geographies and their populations: White European, Yellow Asiatic, Red Aboriginal of the Americas, and Black African (Carter, 2008, p. 88). Hence, Du Bois' (2014): (1) assertion that “the problem of the twentieth century is the problem of the color-line; the relation of the darker to the lighter races of men in Asia and Africa, in America and the islands of the sea”. As we shall see in the upcoming figures for the ensuing racial theories, the older religious or color division evolved, in the twentieth and the twentieth centuries, into a nation(alism) concept which determines the insider-outsider categories (Gorski, 2003; Whitehead and Perry, 2020).

In Figure 1, the duality between the two groups is created by positioning each group based on their individual categorization on the X and Y axes. At the ends of each axis, the categories for color are light/dark and the categories for race are superior/inferior (e.g., see Benedict, 1959, p. 87 on the latter). Then, the White and Black groups are, over time and via struggles and conflicts, slotted into these categories. The progenitors of racial thinking filled out the concepts by allocating White group to the light and superior categories and the Black group (and other groups) to dark and inferior categories such that the White Europeans came out on the top of the racial hierarchy. Inside the graph, one arrow goes from White to Black Americans to show the colorism relation and a parallel arrow shows the racism relation.

Below the graph we have labeled these relationships as racism and indicate that this outcome contrasts a duality between the White and Black groups. Racism is the outcome of the combination of color and race. Racism, in its modern form, refers to the relationship between the groups based on their color-race categorization.^6^ While the White-Black duality has long been theorized as multidimensional, multilevel, and/or a continuum rather than a duality (Christian, 2019), it is still the case that for this paradigm the theorized relationships of racism run in the same direction from White to Black on every dimension at every level. While the alternative—a theorized relationship from Black to White—is also possible under the race paradigm, under this paradigm this would, rather than domination, reflect a politics of liberation. Therefore, the use of more than one dimension, or more than one level, while providing more nuance and assisting in explicating the process through which this relationship operates, does not fundamentally change the underlying relationship presented in this figure. To identify the duality, the research counts the number of arrows directed (or not) at each group. Here White American is 0 and Black American is 2. The duality is not identified by the groups *per se* but by the relationships between them.

**Figure 2:**
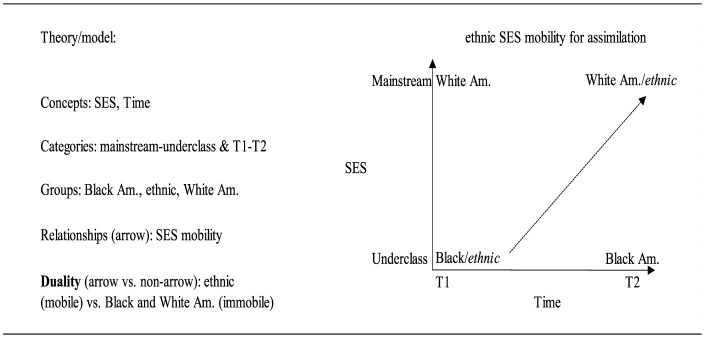
The main components of the original ethnicity (assimilation) duality in embedded relational visualization. T1 and T2 can denote individual mobility as well as multigenerational mobility depending on data operationalization.

Next, in Figure 2 we provide an embedded relational visualization of the ethnic-native or alternatively immigrant-native duality undergirding the original classic assimilation literature. This duality is somewhat more complex because it entails three groups and because it has a dynamic temporal element. Instead of, say, oppressor-oppressed relation and duality, assimilation theory's duality emerges primarily in the form of a mobility vs. a non-mobility.

In Figure 2, there are two concepts, SES and time, that comprise the axes of the graph. The categories of these concepts are, respectively, mainstream/underclass and time 1/time 2. The temporal axis denotes assimilation theories' emphasis that immigrants' upward mobility into the mainstream category is a multigenerational process which unfolds over several decades (Alba, 2024; Alba and Nee, 2003). Assimilation, then, can be thought of in terms of one group moving upwards to join another (cf. Gans, 2007; Luthra et al., 2018 on SES mobility vs. assimilation). In other words, who transforms in relation to whom (Asad, 2024). The interest was in the assimilation of the ethnics or immigrants into the White mainstream (Gordon, 1964; Park, 1928; but see Park, 1914). The idea was that, over time, successive generations of ethnic groups would make various gains in these areas and become more and more similar with the White mainstream eventually becoming part of it —in its original formulation in the first half of the twentieth century, the reference group was the middle-class White Anglo-Saxon Protestant Americans. However, this logic, as well as much of the assimilation theorizing, largely omitted Black Americans as if they are destined to remain stuck at the bottom of society (Wilson, 1987; Metzger, 1971; Jung, 2009; for an exception see Jiménez, 2017). Treitler (2013), to underline assimilation theory's lack of explicitly accounting for Black Americans, uses the metaphor of a chest of racial drawers to show that some groups, such as the ethnics, are theorized to be upwardly mobile across the drawers but that the White-Black racial chest does not change. The inclusion of Black Americans means that the initial duality in assimilation theory in fact entails three rather than two groups, ethnic, White American and Black American. These three groups are then conceptually categorized based on SES and Time.

The relationships here therefore do not delineate relationships between groups at a particular time (though these also exist). Instead, they refer to the difference in position for each of the three groups over time typically via generations. The arrow in the graph delineates mobility for the ethnic group whose relationship refers to ethnicT2 position—ethnicT1 position. By contrast, although there is also a relationship for the White group and also a relationship for the Black group over time, there are no arrows in the graph. This is because neither of these groups have changed their position over time. Hence, the duality is identified by looking at the arrow delineating change over time. There is one arrow for the relationships that indicates mobility and no arrows for the relationships that indicate immobility. Thus, the assimilation duality contrasts a mobile ethnic group(s) with an immobile White-Black or native-born group.

In sum, embedded relational visualizations outline all four components of social theorizing and illustrate the dualities that emerge amongst groups in race and assimilation literatures (in the U.S. context). In the following sections, we apply these blueprints to the theories that have emerged in race and ethnicity paradigms. The particular focus is on Asian and Asian Americans. Not only is the latter the fastest growing minority group (Budiman and Ruiz, 2021), but is also expected to be the largest non-White group by 2055 (Colby and Ortman, 2015). We unpack the evolution of social theorizing to accommodate this new third-group^7^ within race and assimilation paradigms. The underlying thread in these theories has been to surpass dualities so as to maintaining a healthy social balance between groups. Yet, once we apply the embedded relational visualizations, it becomes visible that these triadic social relations reproduce new dualities in the future.

### (Critical) Race paradigm's theories

#### White supremacy theory

It is in relation to the White-Black duality that the problematic of where to position Asian Americans (as well as other groups) arises. Akin to but also different from Black Americans in the U.S., Asians and Asian-Americans have long histories of being racialized under a system of White supremacy (Lee and Sheng, 2023; Omi and Winant, 1994). Historically, Asians were kept out (the 1882 Chinese Exclusion Act), constrained (anti-miscegenation laws and labor practices), and violently interned (Japanese from 1942 to 1945) (Sohoni, 2007; Ngai, 2014). Asians were legally, socially, and physically scapegoated for societal problems including disease and economic downturns (Kwoh, 1993). Accompanying this attack were “Yellow Peril” narratives of Asians and Asian-Americans as predatory and invading (Yang, 2011). Variants of these same tropes have fueled the rising anti-Asian hate tied to the recent COVID-19 pandemic (Li and Nicholson, 2021; Mallapragada, 2021).

At other times, Asian Americans have been stereotyped as culturally “docile”, “stoic”, and “hard-working” model minority (Park, 2008; Chen and Buell, 2018). White Americans used these positive stereotypes to relatively racialize Asian Americans as culturally superior than Black Americans (Kim, 1999). The superior/inferior contrast is then used to pressure Asians to work even harder (as well as deny Asians' minority status) and to critique Black as deficient (e.g., see Chou, 2008; Lee and Zhou, 2015).^8^

**Figure 3:**
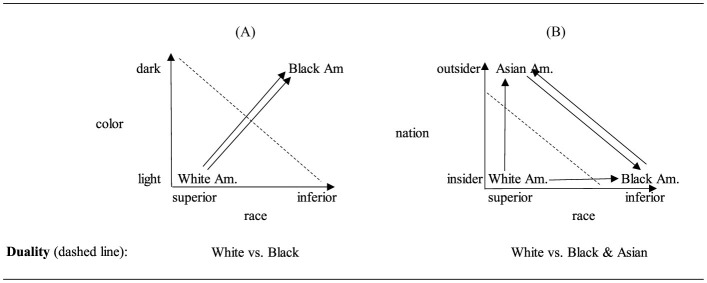
The original **(A)** race duality vs. **(B)** white supremacy theory. Diagram shows a version of White supremacy theory in which Black Am. And Asian Am. are differently racialized. The duality is ascertained by constructing continuums that count the number of arrowheads directed at each group and/or emitted from each group.

The concepts of White supremacy as a system and the racial state were adapted and deployed as a means of bridging across these disparate experiences of race (Bonilla-Silva, 1997, 2005; Feagin, 2006; Omi and Winant, 1994). The idea is that prejudice and discrimination are not simply about individual attitudes and actions but are structural and systemic. Yet the theory isn't simply about social systems, but also about racial groups. Feagin and Elias (2013, p. 947) note, “broadly speaking, we observe in historical and contemporary US data two primary racial projects: that of White Americans who seek to maintain their exploitation, oppression and domination of groups of color, and that of people of color who constantly battle uphill to overcome oppressive and systemic racism”. In effect White supremacy theory draws attention to the different ways that different groups are subject and participate (whether intentionally or not) in racial oppression. Here, in Figures 3A, B we contrast the visualization of the initial White vs. Black duality with the triadic relationship theorized by White supremacy theory.

Figure 3A is the initial White-Black duality. Figure 3B shows the triadic relationship implied by White supremacy theory. The two concepts in this figure differ from the concepts in the racial duality figure. The color concept is replaced with a nation concept designed to reflect the racism of national, political, and civic exclusion of Asian (Americans). The categories of this concept are insider/outsider. The placement of White Americans in the bottom left reflects the theory's group conceptual categorization of White as superior on the race concept and insider on the nation dimension. The placement of Black Americans on bottom right reflects the theory's group conceptual categorization as inferior on the race concept and insider on the nation concept. The placement of Asian Americans at the top left reflects the group conceptual categorization as outsider on the nation dimension and superior on the race dimension. The graph shows that, logically, the only way that Black Americans and Asian Americans can be equally but differentially oppressed by White supremacy is if the arrows between Asian and Black Americans are included. Without these additional arrows, racial oppression would refer to the White-Black relationship only and civic oppression would exclusively refer to the White-Asian relationship.

Finally, the relationships delineated by the arrows between the groups in this figure denote the combination of nation and race concepts best expressed in the idea of racism. The arrows between White and Black Americans and between White and Asian Americans accord with the theory. The challenge for the theory is the meaning of the Asian-Black arrow. According to the logic of the graph and the cross-classification of the concepts, these arrows are also a form of racism whether due to racial or due to national exclusion. If we reframe such relations as internalized racism or internalized White supremacy, then we are changing the dimensional concepts or the outcome concept *post-hoc* to, for instance, internalized racism. The problem is that this new concept is applicable to two of the groups, Asian and Black Americans, but not to the third, White American, and thereby precludes needed theoretical and methodological cross-classifications of the all of the groups. Overall, when the number of arrows directed at the groups is counted, the tally is none pointing at White Americans and two pointing at Asian and Black Americans, respectively. Thus, although there are three social groups, based on the theorized relationship, there are two theoretical or abstract groups: White vs. Asian & Black. The dashed line in the figure further indicates this new duality.

#### Racial triangulation theory

Racial triangulation theory was developed in order to surmount a number of limitations of the existing theories on Asian-Black-White relationship (Kim, 1999; Kim C. J., 2022; Yoon et al., 2024; King, 2010). The theory is “widely taught and cited as the premier explanation of Asian Americans' racial position in the United States” (Wong, 2024, p. 313). Some racial hierarchy theories had added Asian Americans in the middle of the Black-White race duality thereby overlooking the specificity of the Asian American experience (Kim, 1999). Other theories, while emphasizing the ways that different groups of color were differently racialized by white supremacy, dropped or discounted the Asian-Black relationship (Kim, 1999). Racial triangulation theory combines the two. The argument is that, on the racial dimension, White Americans relatively valorize Asian Americans as racially inferior to White Americans but as racially superior to Black Americans. However, on the nation dimension, Asian Americans are excluded in ways that differ from both White and Black Americans. This combination places Asian Americans in the middle or triangulated in a field of racial positions between White and Black Americans.

**Figure 4:**
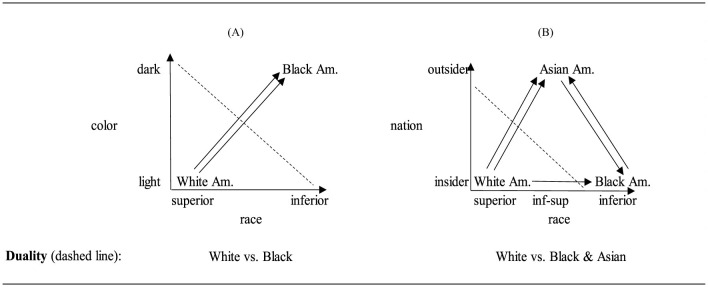
The original **(A)** race duality vs. **(B)** racial triangulation theory.

Empirical and theoretical applications of racial triangulation theory articulate the ways that Asian Americans are positioned between the White and Black (or other minority) groups. Dhingra (2021), for example, describes how high achieving Asian Americans are resented by White Americans for being inferior and are also resented by Black Americans for having surpassed them. Liu's (2018, p. 431) interviewees thought that “Chinese people should stand up for themselves and not to get ‘harassed' or ‘put down by the Americans' anymore”. As Liu explains, this not only means “the white Americans who occupy a superior position in society but also other racial minorities, particularly Black Americans, whose demands seem to be taken more seriously by the state” (Liu, 2018). Fujikane (2022) provides a critical accounting of Asians in Hawaii. They are understood as doubly victimized by the anti-immigrant sentiment of White people over job theft and by Native Hawaiians over land theft. Similarly, in regards to media coverage of anti-Asian violence during Covid-19, Cheng's (2024) finterview participants drew attention to a triangular dynamic in which “‘White media' upholds the framework of the ‘Black attacker' and ‘Asian victims”'. In Figures 4A, B, we visualize the logic of racial triangulation theory. We then explain why this graph is different from Kim's (1999) original graph.

Figure 4A, on the left side, is the initial White-Black duality and Figure 4B shows the triadic relationship theorized by racial triangulation. The two concepts, nation and race, are the same as the visualization of White supremacy theory. The categories on the nation concept are the same and the categories of the race concept are the same except that there is a new middle position labeled as inferior-superior. The placement of the White group in the bottom left reflects the group conceptual categorization of White as superior on the race concept and insider on the nation dimension. The placement of the Black group on the bottom right reflects the group conceptual categorization as inferior on the race concept and insider on the nation concept. The relationships delineated by the arrows between the groups denote the two kinds of racism. In comparison with the White supremacy model, there are now five arrows between the pairs of groups. When these arrows are tallied, there are 0 arrows directed at the White American group, 2 arrows directed at the Black American group, and 3 arrows directed at the Asian American group. Although this numerical count appears to be a ranking, it nevertheless reproduces a White vs. Black & Asian duality since, based on the arrows, both the Black and Asian American groups are numerically closer to one another and similarly distant from the White group.

The Figure 4B for racial triangulation differs from how Kim (1999) had initially imagined the categories, groups, and relations. The direction of the nation and race axes in the original graph was in the opposite direction of the arrows within the graph. Two of the arrows included here—between White-Black on the race dimension and between Black-Asian on the nation dimension—were missing in Kim's original diagram. The visualization adds these missing elements, providing a comprehensive cross-classification of all groups according to the two nation and race concepts. Further, the placement of Asian American in the middle reflects the new categorization of Asian American as outsider on the nation dimension and as inferior-superior (or the middle) on the race dimension. Yet, as motioned above, regardless of amending the visualization, the theory implies that Asian Americans are more disadvantaged than the White and Black American groups: there are no arrows directed at the White American group, two nation and race discrimination arrows are directed at the Black American group, but three nation and race discrimination arrows are directed at the Asian American group. As we discuss in the next section, this count helps to further illuminate why racial triangulation theory was critiqued by the anti-Blackness theory (Sexton, 2010; Davies, 2022).

#### Anti-Blackness theory

More recently, in *Asian-Americans in an Anti-Black World*, Kim (2023) amends her racial triangulation theory and outlines the Asian-Black-White relations so as to further engage with anti-Blackness (see also Aronson and Stohry, 2023; Harpalani, 2021; Matriano et al., 2021 on Asian-Black solidarity and anti-Blackness). In this view, U.S. society is based on a fundamental distinction, not so much between White/Black or White/non-White, but between non-Black and Black (Gordon, 1997; Wilderson, 2003; Sexton, 2010; Yancey, 2003). Anti-Blackness refers to the unique ways that Black people have experienced race and to the ways that other minority groups are implicated in anti-Blackness (Stewart et al., 2023). Kim, taking this argument on board, has developed a newer theorization entailing White supremacy, the racism that contrasts White people with non-White people, and anti-Blackness, the racism that contrasts Black people with non-Black people. To re-visit the earlier theorization of the Asian-Black differences, she writes that, in relation to the White-Black duality, while Asian Americans may not have been White, they were also “not Black” (Kim, 2023, p. 121). The magnitude and scale of racism experienced by the two groups is not equivalent.

**Figure 5:**
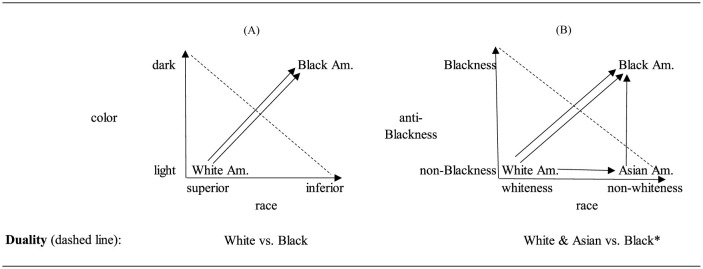
The original **(A)** race duality vs. **(B)** Asian and anti-Blackness theory. ^*^Based on the number of arrowheads Asian Am. is closer to White Am. than to Black Am.

Drawing on archival documents, Kim (2023) contrasts the historical treatment of Chinese immigrants as aliens within the larger project of Black enslavement. She notes that while Chinese immigrants were barred and ejected, this was “from the nation, but not from the Family of Man” (19). Similarly, Wong (2024, p. 319) situates the horrific 1871 lynching of 100 Chinese Americans as taking place within a “broader backdrop of racialized violence against Black people” that includes the lynching of more than 5,000 individuals. Further, addressing the research that presents Asian-American economic and educational success as a myth, Kim (2024) states that this outcome has, at least partially, been facilitated by the latter's non-Blackness. As such, the new approach to social theorizing repositions Asian and Black Americans in very different ways from racial triangulation theory (Pinderhughes, 2024). In Figures 5A, B we provide an embedded relational visualization of the anti-Blackness theory and its focus on both race (i.e., White supremacy) and anti-Blackness.

Figure 5A is the initial White-Black duality and Figure 5B shows the triadic relationship implied by anti-Blackness theory. Here, in line with the new theory, the color and race concepts are replaced with different concepts and categories. The color concept has become an anti-Blackness concept with the categories referencing Blackness and non-Blackness. The race concept remains but with new categories of whiteness and non-Whiteness—as discussed above, such changes imply the reality that these concepts and categories are social constructs and the outcome of social and scientific change. The placement of White American in the bottom left doesn't change from either the White supremacy or the racial triangulation models and reflects the group conceptual categorization of White as affixed to the non-Blackness and Whiteness categories. The placement of Asian American on bottom right reflects the group conceptual categorization as non-White on the race concept and as non-Black on the anti-Blackness concept. The placement of Black American reflects the categorization as Blackness and non-Whiteness.

The relationships, delineated by the arrows between the groups, include four arrows. When these arrows are counted, there are 0 arrows directed at the White American group, 3 arrows directed at the Black American group, and 1 arrow directed at the Asian American group. Accordingly, the new theorization does not simply place Asian American in the middle. Rather it pushes Asian American over to the White American side as there is an even cutpoint or distance between White and Asian vs. Black Americans. The new contrast is White-Asian vs. Black.^9^

### Assimilation paradigm's theories

In the preceding sections, we have reviewed three theorizations of the Asian-Black-White triad: White supremacy, racial triangulation, and anti-Blackness. We showed that first two reproduce a new White vs. Asian and Black duality while the latter results in a Black vs. non-Black duality. In the following paragraphs, we consider three theories from the ethnicity paradigm that have theorized the positionality of Asian immigrants vs. the White-Black duality. We use a slightly modified set of visualizations to show that all three theories reproduce a new duality in the form of SES mobile and immobile groups which, respectively, correspond to an Asian vs. White-Black duality.

#### Segmented assimilation theory

In classic assimilation theory, while theorizing the assimilation of the ethnics into the White mainstream, the idea was that, over time, European ethnic groups would make various socio-economic and cultural gains. As they become more similar with the White mainstream, they would eventually become part of it (Gordon, 1964; Park, 1928). Since the 1980s, segmented assimilation theory shifted the analytical focus to newer immigrants from outside of Europe and to the native-born Black population. Proponents of this model argued that the straight-line assimilation model that applied to early generations of primarily European immigrants no longer held (Gans, 1992; cf. Diaz and Lee, 2023 on ethnics' segmented assimilation; Kazemipur, 2014). Immigrant groups not only assimilate upwards to the mainstream, but can also have two other trajectories. On the one hand, some immigrant youths experience bi-cultural assimilation since they stay within their family and ethnic communities but also find affiliations within the White mainstream institutions. For this group, assimilation is slower than the first group, but ultimately this second group also becomes upwardly mobile over third and further generations (Portes et al., 2005). On the other hand, a third group of second-generations follow a downwards path toward the underclasses of, mainly, Black American and dark skin groups (Portes and Zhou, 1993; Portes and Rumbaut, 2001; Haller et al., 2011). Overall, racialized immigrants experience two main multigenerational outcomes of upward and downward mobility (see the Table in Portes and Rumbaut, 2001, p. 283).

Here the underclass references the Black American group as “an American population whose ancestors, although neither white nor Anglo-Saxon, were Protestants some of whom have been here since before the Declaration of Independence was signed” (Gans, 2007, p. 160). While the model retains the idea from traditional assimilation theory that the upwards pathway is a function of immigrant attainments, the downwards pathway to the underclass is treated as an outcome of racism, bifurcated labor markets, and exposure to oppositional culture found in schools and the inner-city areas (Portes et al., 2005). In terms of empirical results, Zhou and Xiong (2005), for example, find that the patterning of indicators of assimilation such as language, ethnic identification, and sense of belonging, show trajectories that go up and down (or little change) among and within groups such as Vietnamese and Filipino (see also Thomas and Zhou, 2022; Zhou and Yang, 2022 on Chinese immigrants).

**Figure 6:**
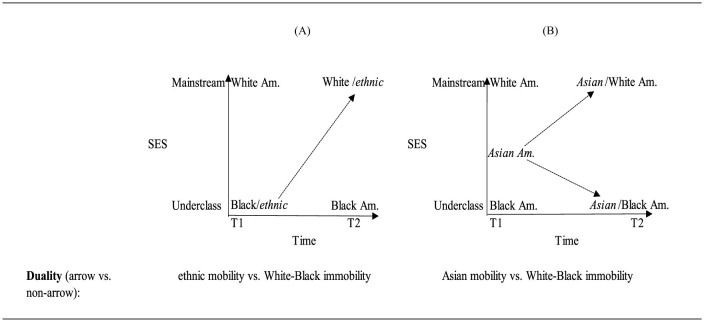
Visualizing the **(A)** original ethnic duality vs. **(B)** segmented assimilation theory.

Figures 6A, B contrast the initial ethnic vs. White-Black duality to the new group positioning and relationships in segmented assimilation theory.

Figure 6A is the initial mobility duality and Figure 6B shows the triadic relationship outlined by segmented assimilation theory. The SES and time concepts are identical to the concepts in the original mobility duality. The categories are also the same. The placement of the Black group and White groups reflects each group's conceptual categorization as, respectively, underclass and mainstream and does not change over time. This is because, according to the theory, White Americans retain their SES and Black Americans linger in the low SES echelon over time. The Asian American group in this theory occupies a different initial position in contrast to ethnics' position in classic assimilation theory. The relationships delineated by the arrows denote the two outcomes that Asian immigrants can take over time. There are no arrows for either the Black American or White American groups since these groups are not theorized as mobile. The comparison between the groups with and without mobility arrows shows that, even though segmented assimilation theory hypothesizes multiple forms of mobility for the Asian immigrant group, the theory does not break from classic assimilation's ethnic vs. White-Black duality. Segmented-assimilation's new form of duality is Asian mobility with White and Black immobility.

#### Ethnic economy theory

In the ethnic (enclave) economy theorization, self-employment, co-ethnic workplaces, and business ownership are theorized as income generating strategies that help immigrants to move out of, or avoid, the underclass (Light et al., 1994; see also Cao, 2022 on Black American economies). This mobility is facilitated by a host of factors including education, social networks, and knowledge of multiple languages and social policies (Gold and Light, 2000). In terms of the relationship between the immigrant group and the underclass, insofar as the middle group is providing an important service (Wong, 1985; Yu, 2022), or because the less-advantaged groups enjoy support from the upwardly mobile ethnic entrepreneurs (Zhou, 2004; Thomas and Zhou, 2022), the relationship between the two groups is thought to be more positive.^10^

To fine-tune the theory, ensuing debate has centered on what, in particular, makes an economy “ethnic” with eight different definitions emphasizing the ethnicity of the entrepreneur, the ethnicity of the employees as well as the geographic context (Pécoud, 2010). The purpose of these definitions is to clarify the boundaries around the concept since if every economy is ethnic, then the concept risks becoming meaningless (Pécoud, 2010). An accompanying discussion has delineated the boundaries of ethnic economies to outline whether they are spatially-delimited or network-based (e.g., see Sharma and Koh, 2019). In terms of geography, there has been an expansion in focus of the ethnic economy as existing in, for example, traditional “Chinatowns” in traditional immigrant-receiving cities to a more recent focus on new immigrant destinations, satellite and ethnoburb economies (Xiong, 2023). Similarly, early work on network linkages, for example, counted the number of Chinese firms in Los Angeles across neighborhoods (Tseng, 1994) whereas more recent scholarship emphasizes the importance of transnational linkages (Thomas and Zhou, 2022).

The ethnic enclave economy is often treated as a response to blocked mobility and discrimination by the dominant native-born group that keep immigrants out of mainstream organizations and occupations (e.g., see Pullés and Lee, 2019). Yet the evidence as to the utility of this strategy is somewhat mixed. At the individual level, a study of the experiences of Korean workers at American and Korean multinational enterprises, found that when, after experiencing discrimination in the mainstream, immigrants “turn to co-ethnic employment out of disappointment and frustration”, they are sometimes faced with new forms of mistreatment (Kim E., 2022). Jang (2021) also found that working in co-ethnic firms lead to worse outcomes for Korean immigrant women.

**Figure 7:**
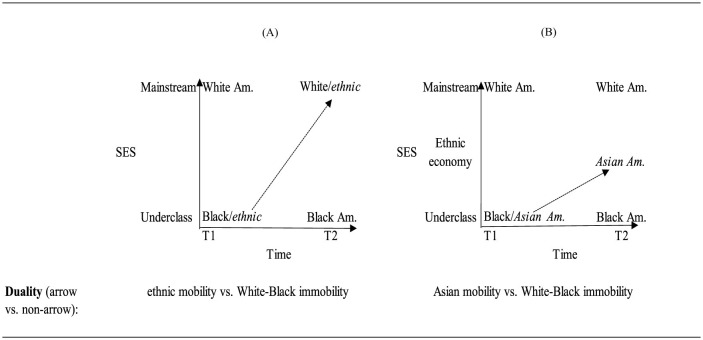
Visualizing the **(A)** original ethnic duality vs. **(B)** ethnic economy theory.

Figure 7A is the initial mobility duality and Figure 7B shows the triadic relationship implied by ethnic economy theory.

In both figures, the SES and time concepts are identical. The categories are also the same. The placement of the Black group and White groups reflects each group's conceptual categorization as underclass and mainstream and does not change over time. The Asian group in this theory occupies the same position as ethnic at T1 in the initial version of assimilation theory. The primary difference is that, rather than moving directly to the mainstream, a middle ethnic economy position is added to the SES concept. The relationships delineated by the arrows denote the trajectories that Asian immigrants can take over time via the ethnic economy (and then with time would eventually join the mainstream). There are no arrows for either Black or White since these groups are not theorized as mobile. Thus, as with segmented assimilation theory the ethnic economy will also reproduce an underlying duality contrasting Asian mobility with White and Black immobility.

#### Neo-assimilation theory

Alba and Nee (1997, p. 863) define assimilation as “the decline, and at its endpoint, the disappearance of an ethnic/racial distinction and the cultural and social differences that express it.” The task of studying assimilation typically often entails a comparison between an immigrant's initial position at one point in time to their position at another point in time (or to a groups' intergenerational mobility) (Marrow, 2013). Among the areas subject to such comparisons are socio-economic status (Kim and Zhao, 2014); language acquisition (Mouw and Xie, 1999), and intermarriage (Alba and Nee, 2003; Qian and Lichter, 2007). Other studies treat individual characteristics such as socio-economic status as predictors of other outcomes such as spatial location (White et al., 1993; Iceland and Scopilliti, 2008; see Schachter, 2016 for a critique of SES is an outcome and as a variable). Some scholars note that, for non-citizens, the lack of citizenship status precludes the acquisition of the socio-economic and other attainments used to denote assimilation (Drouhot and Nee, 2019). Similarly, some Asian refugees did not attain their place of residence they were assigned this location by the state (Waters and Jiménez, 2005). Still, the placement of immigrants on the underclass side is not so much about immigrant agency or lack thereof. Rather, the initial placement of immigrant on one side has to do with who they are not: the reference group which is the non-Hispanic White mainstream.

Alba and Nee (2003) later stressed that, unlike earlier straight-line assimilation theory in which the immigrant group became mainstream (or White), neo-assimilation has a relational component. Over time, the boundary between immigrant and mainstream is blurred thereby altering the character of the mainstream (see also Alba and Duyvendak, 2019). Therefore, it is not simply the minority who changes, the majority also changes; this also changes the overall composition of the groups on each side of the boundary (cf. Alba, 2020 on “decategorization”).

**Figure 8:**
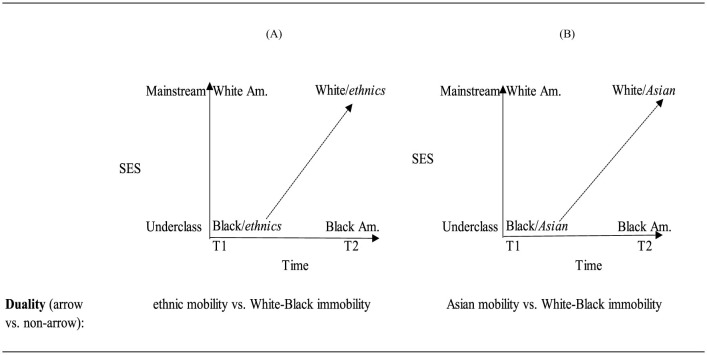
Visualizing the **(A)** original ethnic duality vs. **(B)** neo-assimilation theory.

Considering Asian immigrants, it is acknowledged that may have started as underclass due to their educational and occupational achievement (Neckerman et al., 1999; Suzuki, 1977; Nee and Sanders, 1985), but they have moved up to the mainstream. Within this latter literature, there is a discussion about the mechanisms such as values leading to the groups' attainment and academic and occupational achievement (Ryan and Bauman, 2016). Debates have ensued over the particularities of what is meant by values and culture that have enabled Asians to match the attainments of the mainstream group. Culture has been referred to as Confucian values that prioritize academic aspirations (Huang and Gove, 2015) and work ethics or effort more broadly (Hsin and Xie, 2014; Sakamoto and Kim, 2018). Finally, selectivity arguments either highlight how government policies favor the more advantaged immigrants (Zhou and Lee, 2018) or the mechanisms through which such individuals self-select for immigration (Feliciano and Lanuza, 2017). Regardless of the origins of such differences, these arguments have a common focus on some positive characteristic of the immigrant population as accounting for the differences in outcome. Figure 8A is the initial mobility duality and Figure 8B shows the triadic relationship implied by neo-assimilation theory.

Here the SES and time concepts are identical. The categories are also the same. The placement of the Black and White American groups reflects each group's conceptual categorization as underclass and mainstream and does not change over time. The Asian American group in this theory occupies the same position as ethnic at T1 and T2 as in classic assimilation theory. The relationships delineated by the arrows denote the trajectories that Asian immigrants can take over time. There are no arrows for either Black or White Americans since these groups are not theorized as mobile. Thus, as with classic assimilation theory, neo-assimilation theory reproduces a duality contrasting Asian mobility with White and Black immobility.

## Discussion

The analysis considered the case of Asian Americans and Asian immigrants in relation to a White-Black racial and ethnic duality. The essential argument is that, while the Asian American categorization as neither Black nor White is “post” the old Black or White racial duality, this new duality is not “post” duality writ large. We proposed a new embedded relational method of visualization to help further illustrate and identify these resulting dualities. When Asian Americans and Asian immigrants are situated vis-à-vis the existing White—Black dualities, the net result will be the new White—non-White, Black—non-Black, or Asian—non-Asian dualities. This patterning and the embedded relational method can be further adapted for a multi-axis context (see also Tawa et al., 2013), other groups, quadrants, categories, as well as additional contexts. Still, no matter the change, when the categorization and cross-classification of all three groups on all elements is taken into account, a new duality will emerge.

The visualizations help to show the extent to which the theories operating under the critical race paradigm problematize the group in the superior/or higher “above” position (even if this is not explicit on the two-dimensional plane). That is, even though it is materially better to be in the superior position, the paradigm is such that racism (or the problem) is emanating from the superior group and hence the White side of the White—Black duality (Applebaum, 2010; Bonilla-Silva, 2019; c.f. Iceland and Silver, 2024; Sites, 2025 on epistemic privileging). This problematization adds a built-in incentive to move away from the White group/side and/or the “superior” position. By contrast, assimilation problematizes the group in the inferior or lower “below” position. It is the supposedly inferior group(s) which must “become”, or fail to become, like the supposedly superior group (Karimi and Wilkes, 2023). Thus, the assimilation paradigm implicitly problematizes the underclass (read Black) side of the mainstream-underclass duality (Jung, 2009). Still, as long as Asians Americans and Asian immigrants are below the White American group and/or above the Black American group, then when added as a “third” group there is a logical consistency to who is problematized within each paradigm.

The new challenge for both paradigms is the growing body of recent empirical evidence showing Asian Americans equalling or surpassing White Americans on a host of outcomes including income, education, occupation, health, work ethic, and rates of imprisonment (Bailey et al., 2017; Goyette et al., 2024; Iceland and Hernandez, 2017; Jiménez and Horowitz, 2013; Lee and Sheng, 2023; Sakamoto and Hsu, 2020). While the embedded relational diagrams could be adapted to account for this evidence and the consequent theories surrounding this evidence, the theoretical implications of this evidence are potentially controversial for the theories emanating from each paradigm, albeit for different reasons.

In terms of theories emanating from the critical race paradigm, one response has been to reject this evidence. This rejection might entail dropping Asian Americans from the written or graphical analysis (see Wilkes and Karimi, 2023), invoking other evidence such as heterogeneity within the Asian American population, immigrant selectivity, bamboo ceilings, positive stereotyping and the model minority myth (for critique see Kim, 2024; Sakamoto and Kim, 2018), or making use of alternative measures of Asian American disadvantage entailing only the White group (e.g., see Wu et al., 2021). Another option would be to accept the evidence that a new ranking is developing and to develop theoretical arguments that problematize the middle (read White American) position rather than the top (Asian American) position. Still, no matter which approach is used, the challenge is going to be that such approaches largely invisibilize Black Americans. Empirically, regardless of whether Asian Americans are equal to, above, or below White Americans, the Black-Asian gap is, on most indicators, almost inevitably larger than the White-Asian or Asian-White gap. Theories operating within the critical race perspective might have to adopt more of a Black vs. non-Black or historically marginalized—AsianWhite perspective. This is normatively challenging because, insofar as the paradigm problematizes the group(s) on the non-Black side of the duality, it then problematizes the Asian American group and positionality along with White group and positionality.

The assimilation literature has been more likely to invoke evidence that Asian Americans have equalled or surpassed White Americans (and therefore also Black Americans). While an Asian-White mainstream helps to buttress assimilation theory against charges of upholding implicitly White-centric logics (Jung, 2009), this multiracial mainstream does not resolve the implication that Black Americans must somehow become like the former (see also Karimi and Wilkes, 2023). As with race theories, some research discounts this new evidence by adding new measures of assimilation (Lee and Sheng, 2023) or by questioning whether White and Asian Americans are practically in similar positionalities (Jiménez, 2017). Furthermore, insofar as Asian Americans are above White Americans, then either “the mainstream” is now Asian American and White Americans are pushed down into a “sub-mainstream”, alternatively, White Americans continue to be mainstream and Asian Americans are pushed up into an “uber-mainstream”. Regardless, if this new evidence is to fit with the logic of assimilation theory whereby the groups below should become like the groups above then the implication is that both the White and Black American groups need to become more like Asian Americans.

## Conclusion

It has long been argued that middle categories, new concepts, and other such classificatory options surmount racial and ethnic dualities (Brubaker and Fernández, 2019; Brubaker, 2016; Bynner, 2005; Cornell and Hartmann, 2006; Valdez and Golash-Boza, 2017; Wacquant, 2024). In this paper we used a new embedded relational approach to visualize the theorized relationships of the Asian-Black-White triad as outlined by selected theories from the critical race and assimilation paradigms. In case of the critical race paradigm, we applied these visualizations to three theories—White supremacy, racial triangulation, Asian Americans and anti-Blackness. In case of the assimilation paradigm, we used the embedded relational graphs to visualize the group-specific mobility trajectories for the Asian-Black-White groups as theorized by segmented assimilation, ethnic economy, and neo-assimilation. We showed why, in all cases, each of these theories, when applied to three groups, collapses into a new duality.

The embedded relational visualizations fill in the elements such as the relations, the groups, and the concepts one or more of which is often absent from existing theoretical and/or visual representations of social theories. The visualizations portray the old duality and the three-group relations implied by a particular theory side by side. This visual comparison intuitively illustrates the emerging duality among the three groups. While the addition of new third groups, categories and concepts move us away from the initial dualism, they don't move us away from dualisms writ large. Instead, they merely stretch out the points on the continuum thereby expanding the composition or replacing one of the sides with a new group.

In the case of racial theorizing, we identified the new dualities by considering the number of relationship arrows emitted by and directed at each group. In the case of assimilation theorizing we identified the new dualities by comparing the extent to which groups' over time trajectories indicated mobility or non-mobility. Overall, we identified three possibilities for the emergent dualities: White vs. Asian/Black (White supremacy theory, racial triangulation theory), White/Asian vs. Black (anti-Blackness theory); and White/Black vs. Asian (segmented, ethnic economy, neo-assimilation theory). While this reproduction is “post” the initial duality of White-Black, it is not a “post” duality in the post-structural sense. With three bodies, one of the groups will always be separated from the other two.

The main point of recognizing these dualities and the three-body problem is to detect the limitations of current approaches to racial and assimilation theorizing so as to build better future theories. For instance, the embedded relational graphs show that, in the effervescence of finding and adding new third-groups or concepts to the existing dualities, it is not sufficient to insert the new group in the middle of the duality continuum, binary, or pyramid. The new third-entity must be cross-classified against the existing concepts, categories, and groups and, in turn, be located in the according spot in the graph. Each of the old groups must also be cross-classified in this way including on each of the new concepts. Without such precisions, most racial and assimilations theories, for instance, had included Asian Americans by theorizing the Asian-White relationship (Asian American as oppressed by White American, Asian American as becoming or surpassing White American) while dropping the implications for the Black American group on those same concepts and Black-Asian relations. The embedded relational visualizing method provides a toolkit for race and assimilation theorizing that is aimed at considering the bigger multi-group picture.

## Data Availability

The original contributions presented in the study are included in the article/supplementary material, further inquiries can be directed to the corresponding author.

## Footnotes

^1^Newton's three-body problem remains one of the most challenging puzzles in modern sciences (Musielak and Quarles, 2014). Put simply, the positions and orbits of two gravitationally connected bodies, such as the Earth and Sun, can be accurately predicted, but when a third moving body, such as Jupiter, is introduced, its gravitational force thwarts the Earth-Sun stable circuit. Mathematically, the relation between the two, and now the three, requires new calculations to predict the positionality of each entity in the new emergent gravitational circuit. Either the “gravitational forces could cause two of the three bodies to collide, or they could fling one of the bodies out of the system forever” (Ware, 2024). The main point is that minor changes to the initial starting parameters lead to very different dualistic outcomes: there is no general solution that predicts the composition of the new two-body system (Abdel-Rahman, 2023).

^2^We grant the many other theories within each paradigm including middleman minority (Bonacich, 1973), group threat (Blumer, 1958; Quillian, 1995), tri-racial (Bonilla-Silva, 2005), and racialized incorporation (Chaudhary, 2015).

^3^While Tilly (1998, p. 14) notes that “more complex categorical systems involving multiple religions or various races typically resolve into bounded pairs relating just two categories at a time, as when the coexistence of Muslims, Jews, and Christians resolves into the sets Muslim/Jewish, Muslim/Christian, Jewish/Christian, with each pair having its own distinct set of boundary relations”, he used a logic that solves the problem of how to categorize the third group in relation to a duality by dropping the third group.

^4^A unit of analysis could refer to individuals, groups or alternatively to larger units such as nations.

^5^The White-Black duality traces back to the historical legacy of chattel slavery in the USA as well as the Jim Crow segregation that accompanied the Reconstruction era. Legal classifications of race, based on the “one drop” rule as well a host of discriminatory political and institutional policies perpetuated this duality (Du Bois, 1935; Painter, 2010; Wacquant, 2024).

^6^Despite within group heterogeneity (e.g. see Coburn and Crichlow, 2020 on the multiplicity of Blackness) there is still an inherent underlying duality. In the case of racial theory, the initial duality essentializes the White group and the superordinate or “oppressor-advantaged” position and the Black group as the subordinate or “oppressed-disadvantaged” position.

^7^Asian and Asian American is a heterogeneous group encompassing many smaller groups including Chinese, Japanese, Indian, and Vietnamese (Tran, 2024).

^8^Poon et al. (2016) advocate for an alternative understanding of the model minority myth in which Asian Americans occupy a middleman type of position between the White and Black groups.

^9^Shiao (2017) notes that some Asian Americans adopted by and living in White communities are in effect moved to the White side of a White/Black contrast. Certainly, some scholars take care to note that, although there is an “adjacency” to Whiteness, Asian Americans have not been able to claim membership in this category (Patil, 2021; cf. Sakamoto and Hsu, 2020).

^10^Zhou (2004, p. 1042) writes, “A Chinese immigrant who runs a fast-food takeout restaurant in a Latino-dominant neighborhood is a middleman-minority entrepreneur, but he would become an enclave entrepreneur when he comes back to his other fast-food takeout in Chinatown.” As the outcome and relationship runs in the same theorized direction, this logic entailing Chinese entrepreneurs, Chinese co-ethnics, and Latino ethnics runs into the three-body problem. If the concept is entrepreneurship (yes/no), we can then imagine an arrow running from Chinese entrepreneurs to Chinese co-ethnics and another arrow running from Chinese entrepreneurs to Latino ethnics. Given that the arrows denote the same concept the concept can only be positive or negative across both pairwise contrasts but not positive for one contrast and negative for another
